# How safe are nanoscale metal-organic frameworks?

**DOI:** 10.3389/ftox.2023.1233854

**Published:** 2023-06-23

**Authors:** Dhruv Menon, Swaroop Chakraborty

**Affiliations:** ^1^ Cavendish Laboratory, Department of Physics, University of Cambridge, Cambridge, United Kingdom; ^2^ School of Geography, Earth and Environmental Sciences, The University of Birmingham, Birmingham, United Kingdom

**Keywords:** metal organic framework (MOF), nanosafety, safe by design, machine learning, toxicology

## Abstract

Owing to the size scales that can be accessed, the nanoscale has opened doors to new physical and chemical properties, not seen in the bulk. These properties are leveraged by nanomaterials (NMs) across a plethora of applications. More recently, nanoscale metal-organic frameworks (nMOFs) have witnessed explosive growth due to the modularity of their chemical constituents, the ability to modify their composition and structure, and exceptional properties such as permanent porosity and high surface areas. These properties have prompted the investigation of these materials for applications in biological and environmental contexts. However, one aspect that is often ignored in these discussions is their safety at a nanoscale. In this mini review, we aim to initiate a discussion on the safety and toxicity of nMOFs, drawing parallels with the existing guidelines and literature on the safety of inorganic NMs. We first describe why nMOFs are of considerable interest to the scientific community followed by a discussion on routes through which they can be exposed to the environment and living organisms, particularly shedding light on their transformation mechanisms. The review also discusses the factors affecting toxicity of nMOFs, such as their size, shape, morphology, and composition. We briefly highlight potential mechanisms of toxicity and conclude with describing the need to transition towards data-intensive computational approaches such as machine learning to establish nMOFs as credible materials for their envisioned applications.

## Introduction

**FIGURE 1:**
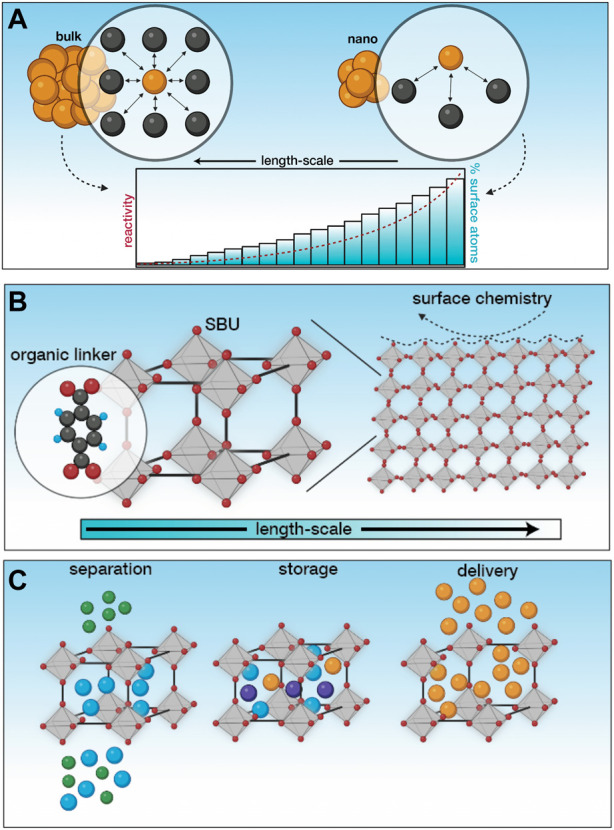
An introduction to nMOFs. **(A)** At the nanoscale, there is a higher proportion of surface atoms leading to higher surface energies, which in turn facilitates higher reactivity. This unlocks exciting physical and chemical phenomena not observed in bulk. **(B)** MOFs are hybrid inorganic-organic crystalline materials composed of secondary building units connected via organic linkers. Owing to the way that they are connected, these materials have high porosity and surface area. Often, the external surface of MOFs are modified through functional molecules to enhance their properties, making them more suitable for the task at hand. **(C)** By virtue of their properties, these materials are used for applications pertaining to separation, storage and delivery, in both biological and environmental contexts. Created using BioRender.

Humanity’s quest towards pushing the boundaries of science has led to the nanoscale, where exciting physical and chemical phenomena have been unlocked owing solely to the size scales that can be accessed (generally <100 nm, however these definitions are arbitrary and often flexible) (Silva, 2004). The observation of these phenomena at these length-scales has not only advanced fundamental understanding of science, but has also served as the basis for emergent applications with far-reaching consequences. These unusual properties can be ascribed to the size of nanomaterials, where they tend to behave more like molecules and less as bulk materials (nanomaterials act as a bridge between the bulk and atomic scale) (Nel et al., 2006). Nanomaterials have a significantly higher proportion of surface atoms than bulk materials. These surface atoms have fewer neighbors than their bulk counterparts, leading to a higher free energy (Figure 1A). Since high free energies are unfavourable, these materials tend to be highly reactive and either bind to other species or undergo specific interactions in order to stabilise themselves (Fruk and Kerbs, 2021).

The engineering of materials at the nanoscale leverages the emergence of these unusual properties across consumer products, electronics and more recently, in medicine (Nel et al., 2006; Valsami-Jones and Lynch, 2015). While the initial focus was on inorganic NMs, a hybrid inorganic-organic class of NMs known as metal-organic frameworks (or porous coordination polymers) have witnessed explosive growth owing to the modularity of their constituents and the ability to modify their composition and structure with relative ease. At the simplest level, these crystalline materials are composed of metallic centers (known as secondary building units) connected via organic linkers using relatively strong bonds (Figure 1B). Owing to the strength of the bonds and the use of relatively long organic molecules, the material formed has significant void space, making them permanently porous (Furukawa et al., 2013). Given the virtually infinite chemical space, a careful selection of MOF building blocks can undergo temperature induced self-assembly that can be modulated to facilitate a fine control over phase purity, porosity, particle size, morphology and surface chemistry (Figure 1B); (Forgan, 2020). In most applications, it is the ultrahigh porosity (*>*90%) and high specific surface area (*>*7,000 m^2^ */*g) that is made use of, for sensing, separation, removal or delivery of specific species (Figure 1C). We have previously used these materials for the removal of toxic metal contaminants (such as Pb(II)) from wastewater (Goyal et al., 2022a; Goyal et al., 2023), which is a very small subset of their applications in biological and environmental contexts. A common observation across applications, however, is the stability of MOFs, which is poor at operating conditions. While the bond between the secondary building units and the linkers are strong enough to allow modulated self-assembly, they are not strong enough to withstand most operating conditions such as moderately high temperatures or interactions with water (Ding et al., 2019). As a result, a third component is often introduced to functionalise the MOF to improve its stability and augment its applicability for the task at hand (Cohen, 2012; Menon and Bhatia, 2022). The flexibility of secondary building units, organic linkers and functional molecules make MOFs incredibly diverse, which is promising from an application standpoint, but makes one process increasingly challenging; assessing and quantifying their safety.

A similar discussion was initiated in the early 2000s for engineered NMs, where there were worries that the environmental impact of NMs would outweigh their benefits, especially since no guidelines were in place to assess them (Colvin, 2003). It was abundantly clear that NMs could not be treated in the same way as bulk materials, as their smaller size gives them additional capabilities such as penetrating cell membranes and causes functional alteration in cells. Several studies point towards a strong correlation between toxicity and physical parameters such as size and shape, and chemical properties such as ability to agglomerate or transform. New phenomena such as the “Trojan horse” are observed at the nanoscale, where nanoparticles are internalised in cells, and dissolve in cellular environments leading to the release of toxic metals inside cells (Valsami-Jones and Lynch, 2015). In a recent study, we tried to observe correlations between a NM’s ability to dissolve and the toxicity it induces. While there was a strong correlation for some NMs, the analysis was much more challenging for others (Chakraborty et al., 2023). Despite the apparent complexities, the nanosafety community has rallied to develop advanced tools for the assessment of the toxic potential of engineered NMs (Fadeel et al., 2018) and there are well-defined regulations and guidelines set in place (Rasmussen et al., 2016). That brings us to an important question; would the same guidelines and regulations serve nanoscale MOFs (nMOFs) well? This review is intended to initiate a discussion on the safety of nMOFs by relating their properties with that of engineered NMs, with a focus on nMOFs physicochemical properties, their impact on biological responses. We also discussed upon the application of computational modelling in making these promising set of materials safer-by-design.

## Parallels between engineered NMs and nMOFs

Several similarities can be drawn between engineered inorganic NMs and nMOFs, primarily with regards to their sizes, shapes, morphology, and intended applications. As mentioned previously, due to high surface energy, these materials are highly reactive, forming more stable phases either through aggregation or dissolution (Casals et al., 2012). From a biological context, this increased reactivity facilitates stronger interactions between these NMs and biomolecules (such as proteins) and machinery (such as cells). It is important to note that it is not the nanoparticle itself that cells interact with, but rather a nanoparticle coated with biomolecules (a “corona”). These proteins associate to the nanoparticle as soon as it is introduced into physiological environments (Lynch and Dawson, 2008; Casals et al., 2010). There exists a very complex interplay between the size of the nanoparticle (due to agglomeration), the rates of binding and unbinding of proteins on the nanoparticle surface during the corona formation, and potential release of metallic ions that would ultimately dictate the interactions of these NMs with cells. Despite their apparent similarities, it is worth noting that the diversities and complexities of engineered NMs pales in comparison to nMOFs. Apart from focusing on physical parameters such as size and shape, one must consider the effect of virtually infinite individual nMOF pre-cursors and their interactions with their immediate environment. This inherent diversity forces a need to go “above-and-beyond” standard nanosafety protocols. Nonetheless, there are several lessons that can be learnt from the work on engineered NMs, and several parallels can be drawn as we navigate the nanoscale MOF landscape.

Toxicity at the nanoscale affects both ecosystems and living organisms. Once released into the environment, nanoparticles tend to interact with the air, soil and water (Elsaesser and Howard, 2012). The ecotoxicity of NMs is most often studied on aquatic organisms, with several correlations drawn to mammalian studies. Taking note of lung damage during studies on mammals, injury to the gills and intestine were predicted for aquatic organisms upon exposure, due to the presence of similar epithelial tissues. Similar studies have since been conducted for assessing the fate, transformation and toxicity of nMOFs when exposed to aquatic organisms such as *Daphnia magna* (Li and Wang, 2022) and *C. elegans* (Goyal et al., 2022b). Commonly studied exposure routes were aqueous exposure (Handy et al., 2008), natural feeding mechanisms (Goyal et al., 2022b) and trophic transfer. (Li and Wang, 2022). In these cases, there are concerns of long-term health implications even after short exposure, albeit at higher concentrations likely only during accidental spills (Handy et al., 2008).

The behaviour of inorganic NMs and nMOFs in colloidal dispersions are critical towards understanding their fate, transformation and resultant toxicity. Due to particle-particle collisions, dispersions are thermodynamically unstable, tending to aggregate and separate. The rate of aggregation is higher for particles of different sizes (polydisperse) and have implications on their interactions with organisms (Handy et al., 2008). Several theories such as Derjaguin and Landau (Derjaguin and Landau, 1941), and Verwey et al. (1948) (DLVO) account for attractive and repulsive interactions between closely adjacent particles. In-depth insights of these theories and their shortcomings in predicting the behaviour of nanoparticles in the environment has been discussed by Handy et al. (2008).

With regards to exposure from a biological context, nMOFs can be treated akin to engineered NMs due to similarities in size (dimensions of the order of 100 nm) and the applications for which they are employed. Here it is important to differentiate between engineered NMs and ultrafine polydispersed particulates. In a seminal communication on nanomaterial toxicity, Colvin argues that engineered NMs in general should not be compared to these particulates. This is because particulates are generated as aerosols, and the most likely exposure route is through the same medium. Since aerosols are highly dilute, agglomeration is not commonly observed. In contrast, engineered NMs and nMOFs alike, are most usually synthesized in a liquid phase, and are used for applications in a solid state. In these phases, the concentrations are high enough to allow particle-particle interactions leading to the formation of aggregates which behave very differently during exposure and uptake (Colvin, 2003).

**FIGURE 2:**
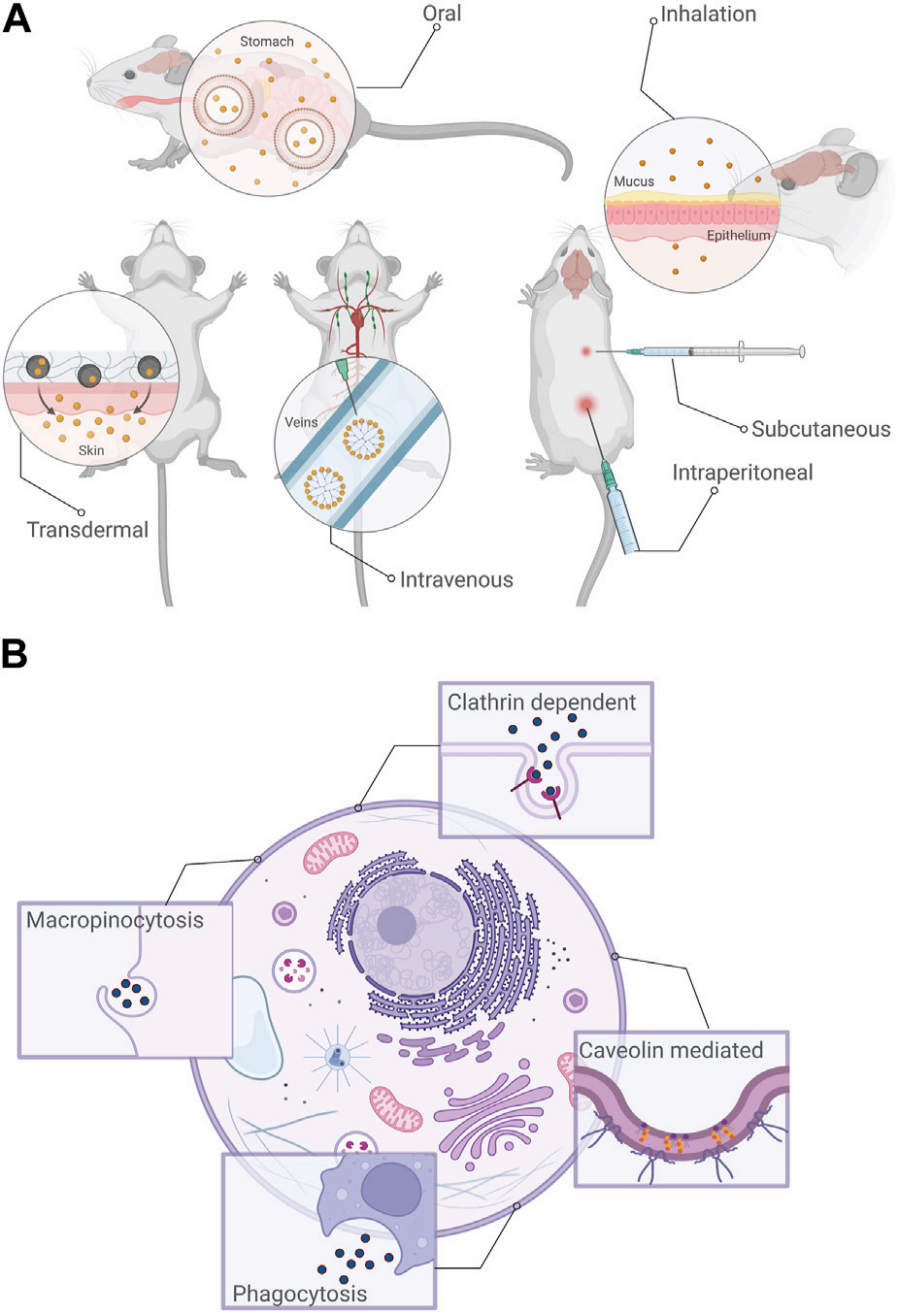
**(A)** Common routes of exposure to nMOFs. Depending upon the applications for which they are used, nMOF exposure to living organisms would be most likely through intravenous, transdermal, subcutaneous, inhalation, intraperitoneal or oral routes. **(B)** Routes of endocytosis most commonly reported for nMOFs: Clathrin-dependent, Caveolin mediated, phagocytosis and macropinocytosis. Created using BioRender.

The most likely cause of human exposure to nMOFs is intentional, i.e., nMOFs being used as carriers for biomedical applications such as drug delivery, biosensing or bioimaging (Menon and Bhatia, 2022). It is important to understand that nMOFs are being used as carriers to improve the biodistribution of the therapeutic agents for enhanced targeting of diseased cells (Simon-Yarza et al., 2018). In the context of their applications, there are six possible routes for the exposure to nMOFs (as is the case with NMs being used for biomedical applications): intravenous (within a vein), transdermal (across the skin), subcutaneous (through the fatty tissues below the skin), inhalation, intraperitoneal (within the peritoneal cavity) and oral (Simon-Yarza et al., 2018); (Sharifi et al., 2012) Among these exposure routes, inhalation, ingestion, contact with the skin and intravenous injection are more likely, again depending on the application (Figure 2A); (Sharifi et al., 2012). For example, if the nanoscale MOFs are being used for environmental remediation applications, the likely exposure routes would be inhalation, ingestion or through skin contact, whereas for biomedical applications, intravenous injection, ingestion or inhalation are more likely.

Post-exposure, the interactions of the NMs with blood is critical towards understanding its fate and resultant toxicity. A lack of compatibility with blood leads to the formation of clots or blood coagulation due to adsorption of proteins onto the NMs surface in order to minimise surface energies. NMs surface gets rapidly coated with specific proteins present in the blood leading to the formation of an entity known as the “protein corona.” In case the nanomaterial has to overcome additional physiological barriers before entering the blood stream, it adsorbs additional biomolecules (Sharifi et al., 2012); (Cedervall et al., 2007). The entity formed post adsorption of the relevant biomolecules dictates the interaction of the nanomaterial with cells, and the resulting fate and biodistribution in the body. Interestingly, in the case of MOFs, this phenomenon (sometimes referred to as opsonisation) often rendered them unusable for drug delivery-based applications. As a solution, studies used functional molecules such as polyethylene glycol (PEG) to render MOFs undetectable to these proteins (Chen et al., 2021). Unfortunately studies pertaining to the adsorption of biomolecules onto the surface of nMOFs, their biodistribution and metabolism are sparse.

From the literature that does exist, it is observed that nMOFs tend to aggregate and accumulate in the lungs, which make them good candidates for drug delivery to the lungs, but also raises concerns of respiratory toxicity (Simon-Yarza et al., 2018); (Baati et al., 2013). Specifically with regards to engineered NMs, nMOFs have three specific advantages for pulmonary drug delivery as highlighted by Zheng et al. (2022), (a) a richer elemental composition and variable porosity allowing diverse structures with varying attributes (b) high void space allowing rapid diffusion of high amounts of drugs (c) the ability to post-synthetically modify the outer surface. With regards to bio-compatibility however, while many studies do highlight favourable outcomes (increased bio-compatibility), most of them focus on *in vivo* or *ex vivo* studies. Studies at both a molecular level and epidemiological level are yet to be conducted, making it difficult to draw clear conclusions.

Additionally, reversible accumulation is observed in the liver and the spleen, and depending on the chemistry of the linker, in some cases, slight accumulation is also observed in the brain. Interestingly, the biodistribution changes upon loading the nMOFs with drugs, making it difficult to extrapolate distribution profiles. These profiles also change depending on the guest molecule that is loaded (Simon-Yarza et al., 2018). A better understanding of these phenomena are crucial before these materials can be considered as serious candidates for biological applications at a clinical level. Simon-Yarza et al. (2018) provide a framework to gauge the suitability of a platform from a safety perspective for drug delivery applications.

## Factors affecting the toxicity of nMOFs

When it comes to the factors that affect the toxicity of nMOFs, the discussion tends to diverge from conventional wisdom surrounding engineered NMs, due to the inorganic-organic crystalline nature of the former. An obvious starting point for these discussions would be the inherent toxicity of the building blocks. When it comes to the inorganic metallic cluster, the commonly used metallic centers are Al, Co, Cr, Cu, Fe, Mg, Mn, Ni, Zn, and Zr, among which MOFs containing Al, Co, Cr, and Fe have relatively lower toxicity, Mg, Ni, Zn, and Zr have moderate toxicity while Cu and Mn have relatively high toxicity. These observations point towards an inverse correlation between the stability of the MOFs in biological media and toxicity, implying that a higher release of metallic ions leads to a higher toxicity. However, this correlation is not linear as one might expect. Similarly, when it comes to the organic linker, the inherent toxicity does seem to have a correlation with the resulting toxicity of the MOF. Factors such as the hydrophobicity of the organic linker dictates the rate of clearance from the body. While the inherent toxicity of building blocks does not necessarily paint a clear picture of the toxicity of the MOF, as a general rule of thumb, the lower the toxicity of the precursors, the lower the toxicity of the MOF (Baati et al., 2013); (Ruyra et al., 2015) As pointed out earlier, the introduction of a functional molecule adds a third order of complexity to the mix, as it could either accelerate or decelerate the release of the metallic center into the biological media and could either be in compliance with or counteract the effect of the organic linker. In the context of biological applications, nMOFs are usually functionalized using nucleic acids, lipids, aptamers, and enzymes to either maintain or improve bio-compatibility while enhancing applicability (Menon and Bhatia, 2022). For environmental applications, the scope of functionalization is much broader, as there are no constraints such as bio-compatibility. Functionalization is carried out either during synthesis or post-synthesis depending on the reaction conditions involved and the strength of the interaction required between the functional molecule and the nMOF. That being said, most applications rely on post-synthetic modifications. Post-synthetic modifications can further be broken down into two broad categories of covalent and non-covalent functionalization, depending on the type of interaction, relative strength and ease of assembly. For a more detailed discussion on methods of functionalization, readers are directed to Cohen (Cohen, 2012).

Considering the building blocks as the first layer of complexity, the physicochemical properties of the material can be visualised as the second layer. Consider the size of the MOF; as size decreases, the ability of the MOF to penetrate physiological barriers such as cell membranes and the blood-brain barrier increases significantly, which in turn, increases its ability to cause severe damage at a cellular level (Baati et al., 2013); (Ettlinger et al., 2022). However, the analysis is not so straightforward, as at smaller sizes (as discussed earlier), surface energies are so high that particles tend to agglomerate to stabilise, which in turn leads to a more rapid clearance from the system. When it comes to the shape of the MOF on the other hand, Wuttke and others (Ettlinger et al., 2022) argue that shape or topology might not have a major role to play in overall toxicity, citing a lack of evidence to the contrary. This is particularly surprising, as shape does tend to have a sizable contribution to toxicity in the case of a wide-array of engineered NMs (Aillon et al., 2009).

A majority of the applications of MOFs rely on their remarkable surface properties, which implies that surface chemistry controls a majority of the interactions. The ability to control the surface chemistry of MOFs thus presents itself as a promising route towards controlling its toxicity, and applicability (McGuire and Forgan, 2015). All put together, there seems to be a complex interplay between the inherent toxicity of the metallic secondary building unit, organic linker, functional molecule, the size, surface chemistry, and colloidal stability of the MOF. Understandably, experimental approaches towards the optimisation of MOFs both from an application and toxicity standpoint seem to be increasingly complex.

## Potential mechanisms of toxicity

The trafficking of species of biological interest into the cell through highly controlled and regulated mechanisms is formally termed “endocytosis”. In the context of cellular function, endocytosis regulates nutrient uptake and cell signaling among other crucial tasks. In nMOFs, it is this mechanism (or rather set of mechanisms) that regulate their internalization whether for intended applications (such as drug delivery or bioimaging) or unintended/accidental exposure. (Linnane et al., 2022). In the simplest possible terms, when nMOFs reach the cellular environment, they interact with the extracellular matrix and the cell membrane. Membrane invaginations engulf the material, leading to the formation of vesicles which are then transported through specialised compartments into the cell (Figure 2B). Based on the specific mechanism, the processes can be described as clathrin-mediated, calveolin-mediated, cathrin/calveolin independent, phagocytosis and macropinocytosis (Behzadi et al., 2017). While an in-depth discussion of these pathways is outside the scope of this review, briefly, most mechanisms are classified on the basis of the proteins involved in the trafficking process. In the case of clathrin-mediated endocytosis, the protein clathrin forms pits along the cell membrane which through a scission process forms vesicles that are trafficked inside the cell. Caveolin-mediated endocytosis involves the protein caveolin forming an invagination along the cell membrane. Clathrin-/caveolin-independent pathways involve other specialised proteins, macropinocytosis involves endocytosis of materials that are soluble and phagocytosis involves the internalization of larger particles (Figure 2B); (Linnane et al., 2022).

Among mechanisms of toxicity, one that is very commonly observed in the context of nMOFs (and engineered NMs in general) is the generation of excessive reactive oxygen species (ROS). ROS are oxygen radicals, with one or more unpaired electrons, for example, superoxide (O^*−*^), hydroxyl (OH^*−*^), hydroperoxyl (HO^*−*^), and certain oxidizing agents such as hydrogen peroxide (H_2_O_2_) and ozone (O_3_) (Bayr, 2005). In the presence of redox-active metal (released from MOFs), there is a steep increase in the concentration of these species (much high than normal physiological conditions) leading to an increased oxidative stress. This increase in stress in turn leads to DNA/RNA damage, modifications of proteins and oxidation of lipid membrane constituents, having a major effect on cell viability (Ettlinger et al., 2022). It should be noted that in most cases, the toxicity of the organic linker and functional molecules are not well explored or explained, presenting itself as a critical bottleneck for establishing standard protocols for the testing of nMOFs.

## Towards a computational future

A key takeaway from the previous discussions is that safety-by-design is particularly challenging in the context of nMOFs owing to the sheer diversity of pre-cursors. It is evident that there is a complex interplay between several factors such as the chemistry of the individual building blocks, strength of interactions, shape and size, porosity and morphology, among several other features. Unfortunately, this suggests that an understanding of the potential toxicity of the individual building blocks does not necessarily dictate the toxicity of the resulting MOF. This also implies that relying purely on experimental approaches would be unfeasible owing to the associated timescales, costs and resources. This sets the stage for transitioning towards a computational future.

Given that fundamentally, MOFs are composed of organic and inorganic pre-cursors, in theory it is possible to leverage the well-established chemistry of each component to gain insights into the molecular and solid-state chemistry of MOFs. In a review by Mancuso et al. (2020), the authors suggest that in a majority of cases, MOFs can be considered to be an array of ordered molecules (treated as a sum of their parts). This simplification has allowed density functional theory (DFT) to make major in-roads into the MOF ecosystem. DFT is used for the electronic structure modelling of MOFs, which can be used to calculate the energetics of MOF interactions with the environment. For example, in a previous study, we leveraged DFT calculations to compare the interactions of different MOFs with water, allowing us to gauge their suitability for toxic metal removal from water (Goyal et al., 2023). In the context of toxicity and safety, DFT in particular can be used to analyse dissolution and degradation when subjected to operating conditions, which as we discussed earlier, majorly contributes to toxicity. Another set of computational approaches are molecular dynamics (MD) and grand canonical Monte Carlo (GCMC) simulations, which allow the exploration of larger length-scales and timescales as compared to DFT. These techniques are better suited for assessing the suitability of MOFs for adsorption, storage and separation-based applications (Bernini et al., 2014).

Despite these promises, the computational costs and timescales associated with the methods discussed above make them unsuitable for large-scale chemical space exploration and screening. This is where, given ample amounts of “good” data, machine learning comes to the rescue. In the context of MOFs, we envision that machine learning can make in-roads in three ways: (I) screening large databases to identify ideal MOF candidates with low toxicity for the application at hand. Researchers have previously had success in using these techniques for the screening of MOFs for storage applications (Borboudakis et al., 2017). (II) For the prediction of MOF toxicity (we however acknowledge that the available data is sparse and biased, making this more challenging to tackle). Researchers have previously been able to develop accurate predictive algorithms for the water stability (Batra et al., 2020), pore guest accessibility (Pétuya et al., 2022) and adsorption (Pardakhti et al., 2017). (III) The more ambitious, inverse design of low toxicity MOF candidates for the application at hand. Generative modelling has only recently been introduced in the context of materials science (Menon and Ranganathan, 2022), and preliminary work on MOFs has begun (Yao et al., 2021), (Kim et al., 2020) As a word of caution however, it is important to have a fundamental understanding of the working of machine learning models, good quality data and accurate checks and balances in place, to get reliable, meaningful and useful outputs from these models.

## Conclusion and perspectives

From an application point-of-view, it is evident that nMOFs have the potential to be useful at a large-scale across biological and environmental applications. The existing literature on nMOFs raise concerns of their adverse effects if present at significant concentrations. There exist significant knowledge gaps both in the ecotoxicology and biological toxicology of these materials, that stand in the way of large-scale adoption. It is therefore time that the MOF community begins to ponder about regulations and standards when it comes to their safety. Luckily, the community does not have to start from scratch, but rather build on the foundations set by the decades of work done on engineered NMs. Given the immense compositional diversity of MOFs, it is necessary to go above and beyond the state-of-the-art for engineered NMs.

To understand the toxicology of nMOFs, it is crucial to understand their biochemical transformation and reactivities. In environmental contexts, this refers to phenomenon such as agglomeration, dissolution, sedimentation, speciation and precipitation while in biological contexts, this refers to formation of biomolecular corona, intra/extracellular dissolution and manymore. In either case, not just the pristine nMOFs, their transformed form could interact with organisms, leading to biological responses. For biomedical applications such as drug delivery, considerations must be given to the route of nMOF administration, dosage, biodistribution and pharmacokinetic profiles. As is the case with inorganic NMs, the physicochemical properties are very crucial for understanding the interactions of nanoparticles with biological entities. In the case of nMOFs, the problem is made more complex due to the presence of guest molecules and functional molecules that have a significant impact on the resultant properties and thus, interactions. Given the compositional diversity, it is evident that relying solely on experimental approaches is unfeasible, and the focus should transition towards an increased dependence on computational approaches. Through community-driven efforts, a large-scale responsible adoption of computational methods such as machine learning, would go a long way towards realizing the true potential of these materials.
